# Assessment of Airborne Bacterial and Fungal Communities in Selected Areas of Teaching Hospital, Kandy, Sri Lanka

**DOI:** 10.1155/2019/7393926

**Published:** 2019-06-12

**Authors:** Premina Sivagnanasundaram, R. W. K. Amarasekara, R. M. D. Madegedara, Anuradha Ekanayake, D. N. Magana-Arachchi

**Affiliations:** ^1^National Institute of Fundamental Studies, Kandy, Sri Lanka; ^2^Respiratory Diseases Unit, Teaching Hospital, Kandy, Sri Lanka

## Abstract

Nosocomial infections, in lay term known as hospital acquired infections, are caused mainly by airborne pathogens found in healthcare facilities and their surroundings. The aim of this study was to quantify and identify bacteria and fungi in a hospital, which is an understudied area of air quality in Sri Lanka. Air samples were collected in agar medium and petri plates containing sterile filter papers. The number of culturable and total airborne microorganisms was estimated by manual counting and fluorescent microscopy, respectively. The morphologically distant bacteria and fungi were identified by DNA sequencing. The statistical analysis revealed significant variances between studied sites (*p* < 0.05) where Outpatients Department and Respiratory Unit showed higher levels of airborne microbial load. Culturable microbial count was higher at noon (hospital visiting hours) compared to other sampling periods (after hospital visiting hours) within the hospital. Total count of airborne microbes was found to be the highest during the afternoon. The most sensitive zones such as Operating Theatre and Intensive Care Unit showed considerably higher counts of airborne microbes. Identification by molecular means revealed the presence of human pathogens in the hospital air including* Bacillus *sp,* Micrococcus *sp,* Pseudomonas *sp,* Staphylococcu s*sp,* Exiguobacterium *sp,* Enterobacter *sp,* Escherichia *sp,* Sphingomonas *sp,* Massilia *sp,* Kocuria *sp,* Fusarium *sp, and* Aspergillus* sp. In conclusion, the results from this study indicate that the hospital air was generally contaminated. Therefore, the implementation of proactive policies and strategies are needed to monitor hospital air quality in sensitive zones as well as other areas of the hospitals.

## 1. Introduction

Air is the prime cause of life on earth. However, air pollution is becoming a serious threat to the continuation of biotic and abiotic factors on the earth. The air is a mixture of gases of different proportions and dusts. Industrialization is a major responsible factor for air pollution which makes the air quality poor in terms of chemical composition. But the biological aspect of air quality does not get enough attention compared to chemical aspect. Biological pollutants or bioaerosols pose equal or more severe threats than chemical pollutants. Bioaerosols originated from bacteria, fungi, virus, and parasites can be hazardous to human health as they pose an ability to remain suspended in the air for extended periods of time and the time between the exposure and damage to the life is usually very short. Indoor air pollution is a major responsible factor for severe illnesses and deaths compared to outdoor air pollution, as human beings spend major part of their time indoors. Indoor air pollution has caused approximately 2 million deaths in developing countries and has been found to be responsible for 4% of the global disease burden [1].

The biological air quality varies between different settings. Healthcare settings face direct and inevitable threats from infectious bioaerosols. Infections acquired in healthcare facilities have been listed as one of the major causes of death and increased morbidity among hospitalized patients [2]. Hospital acquired infection (HAI) is defined as an infection acquired by patients during a short or prolonged hospital stay. The HAIs are responsible for not only significant morbidity and mortality, but socioeconomic burden to the affected families as well. The development and frequency of HAIs are influenced by several factors which can be categorized under three major factors: microbial agents, susceptibility of patient, and environmental factors.

Therefore, a constant surveillance ought to be maintained to minimize the contamination level in the air. Since developing countries including Sri Lanka have very limited facilities and modalities to achieve this target, frequent assessment and monitoring of air quality is very important to improve safety measures. Though several research studies have been carried out to assess indoor air quality, only few studies are available with regard to the healthcare sector and less so in Sri Lanka.

According to some Sri Lankan studies, the ambient and indoor air quality in Sri Lanka is a major problem and the air pollution can be considered as a neglected public health problem in the country [3–5]. The present study investigates the air quality within the premises of Teaching Hospital, Kandy, the second largest medical institution in Sri Lanka built in 1861. It provides secondary and tertiary medical services to the general public in addition to teaching, training, and research opportunities in the Central Province of Sri Lanka.

This study was designed with an aim of assessing the quality of indoor air with regard to microbes in a healthcare facility in Sri Lanka. The objectives of the study were to quantify the total and culturable counts of aerosolized microbes in selected sites and to identify airborne bacterial and fungal species. The abstract of this article was presented before at PGIS Research Congress 2017-University of Peradeniya, Kandy, Sri Lanka.

## 2. Materials and Methods

*(i) Sampling Sites and Periods.* Seven sites in Kandy Teaching Hospital were selected as study sites which included Outpatients Department (OPD), Surgical Intensive Care Unit (ICU), Theatre in Gynecology and Obstetrics Department (TH), and four sites in the respiratory diseases unit: female ward (RF), male ward (RM), bronchoscopy unit (RB), and medical officers' room (RO). A laboratory at NIFS/Kandy was selected as a control site (CON). The respiratory diseases unit is located facing a main road in the city. Both female and male wards were big with windows; however they lacked exhaust fans while bronchoscopy unit and medical officers' staff room were very small in size and fully enclosed and seemingly had old air conditioning system. Surgical ICU and Theatre were fully equipped with air conditioning systems and were large enough. The OPD is large and had more open areas to the environment. During the sampling the bronchoscopy unit and Theatre were active few times with bronchoscopy procedure and surgeries including C-section were being carried out, respectively. The female and male wards of the respiratory diseases unit consisted of around 20 patients at each ward with various respiratory diseases including tuberculosis and lung cancer. The control room which is a laboratory is used for routine microbiology research. Respiratory diseases unit is cleaned by sweeping and mopping the floor daily while OPD is cleaned by mopping the floor once in every two days and daily sweeping. The disinfection procedures of Surgical ICU and Theatre include mopping the floor twice daily and wiping the instruments and all the equipment with surgical spirit. The construction of buildings in the hospital coincided with the sampling period near some of the sampling sites such as Operating theatre and Surgical ICU.

Visiting hours at the hospital include 6.00 am–7.00 am, Noon 12.00–1.00 pm, and 5.00 pm–6.00 pm. Sampling was done during the periods of morning (9.00 am–10.00 am), noon (12.00–1.00 pm), afternoon (2.00 pm–3.00 pm), and evening (5.00 pm–6.00 pm) in the months of January–April, 2017, covering “during” and “after” visiting hours of the hospital between 9.00 am and 6.00 pm. 

*(ii) Sampling and Cultivation of Airborne Microbes.* The passive sampling based on settle plate (gravitational sedimentation sampling) method was used to collect air borne particles containing microorganisms. Air samples were collected in Luria Bertani (Alfa Aesar) or Nutrient Agar (HIMEDIA) plates by direct impaction and petri plates containing sterile Whatman  ^Tm^ No 5 filter papers (with an area of 0.0057 m^2^) with a sampling duration of 30 minutes (1 m^2^ area of the filter paper or the agar plate represents 1 m^2^ area of surface contamination settling from the air at respective hospital sampling site). All the plates were kept on nearly clean areas such as on top of the filing cabinets or tables to capture the airborne microbes in breathing zone. Temperature and total number of individuals at each site were monitored simultaneously during the sampling. Each sampling with respect to time period was done in triplicate and each time the plates were kept in duplicate. Agar plates were incubated at 25°C (48 hours–72 hours). Plates with filter paper were subjected to enumeration of total airborne microbes.

*(iii) Estimation of Total Number of Airborne Microbes (Culturable and Nonculturable-Live and Dead). *Filter papers on the agar plates were used to prepare filter paper suspensions by eluting all the deposited microbial cells to Milli-Q water medium. It was achieved by cutting the filter papers into small pieces and soaking in 10 ml sterile Milli-Q water followed by shaking in an orbital shaker (ORBITEK) for 1 hour at 95 rpm. Filtered suspension was subjected to the next step.

Nucleic acid gel stain SYBR® Green 1 (1X) was used to stain microbial cells (both live and dead cells) in the filter paper suspension for 15 minutes under dark condition. After incubation, the sample was transferred to a clean gridded Sedgewick Rafter counting cell (Wildco 1801-G20, USA) with total volume up to 1000 *μ*l (each small square represents 1 *μ*l volume) and a clean cover slip was placed on it. The negative control setup was carried out using an autoclaved filter paper. An inverted microscope with fluorescence upgrading capability (Olympus CKX41) was used for manual counting of microbial cells. The total number of green coloured round shaped individual cells was counted against a black background. The average cell count obtained for a field (1*μ*l) was extrapolated to the entire sample field (1000 *μ*l). The final count was obtained as number of cells/m^2^ by the following equation.

Area of a filter paper – 0.0057 m^2^.

Total cells deposited/settled on 0.0057 m^2^ area of filter paper (10 ml of suspension) = total number of bioaerosols settled from 0.0057 m^2^ area of hospital setting:

(1) Total  number  of  bioaerosol  cells  settled  from  1  m2  area  of  hospital  setting=10×Total  number  of  cells  in  1ml  of  Milli-Q  water  with  SYBR  Green  10.0057  m2

*(iv) Culturable Microbial Sample Analysis.* The number of both bacterial and fungal colonies was counted on each agar plate after incubation and the count was obtained as colony forming units per m^2^ area (cfu/m^2^). Morphology of each colony was noted with respect to pigmentation, margin, shape, elevation, and other characters and morphologically different colonies were subcultured on LB agar or NA medium until a pure colony was obtained. Pure colonies were stored in sterile microcentrifuge tubes containing LB broth at -20°C until further use. 

*(v) Identification of Isolated Colonies.* The bacterial isolates were identified either gram positive or gram negative based on their reaction with gram stain [6]. Morphology of each colony was noted in terms of pigmentation, margin, size, shape, elevation, surface texture, and appearance. Twenty-four morphologically different cultures of bacteria and five cultures of fungi were further identified by molecular techniques. Genomic DNA extraction was carried out using modified CTAB (cetyltrimethylammonium bromide) method [7]. 

*(vi) PCR Amplification of Purified Bacterial DNA.* 16S rRNA gene region of bacterial genomic DNA was amplified using universal bacterial primers. Each of the PCR reaction systems contained 2 *μ*l of Forward primer (0.4 *μ*M), 5'-AGR GTT TGA TCM TGG CTC AG-3', 2 *μ*l of Reverse primer (0.4 *μ*M), 5'-GGY TAC CTT GTT ACG ACT T-3', 5 *μ*l of PCR Green buffer (1X), 1.5 *μ*l of MgCl_2_ (1.5 mM), 2.5 *μ*l of dNTP (0.1 mM), 0.2 *μ*l of Taq DNA Polymerase (1 unit), and approximately 7.5 ng of DNA template. It was followed by the addition of 20 *μ*l of mineral oil on top of each PCR reaction mixture. The* E. coli* ATTC 25922 DNA was used as the positive control and PCR master mix with Milli-Q water was used as a negative control.

*(vii) PCR Amplification of Purified Fungal DNA.* The internal transcribed spacer (ITS) region in the 18S rRNA gene of fungal genomic DNA was amplified using universal fungal primer pair ITS 5/ ITS 4. Each of the PCR reaction systems contained the same components as in the bacterial DNA amplification and different set of primers which were the following: Forward primer ITS 5 (5′-GGAAGTAAAAGTCGTAACAAGG) and Reverse primer ITS 4 (5′-TCCTCCGCTTATTGATATGC). The PCR master mix with MilliQ water was used as negative control. 

*(viii) Isolation of PCR Products and DNA Sequencing.* The amplified PCR products and controls along with molecular markers (Promega): 2 *μ*l of 1kb DNA ladder and 2 *μ*l of 100 bp DNA ladder for bacterial and fungal DNA, respectively, were electrophoresed on 2% ultrapure agarose gel stained with ethidium bromide at a constant voltage of 110V and followed by visual examination on gel documentation system (SYNGENE–G:BOX).

The DNA was extracted from the gel according to manufacturer's protocol using a DNA Purification Kit (Promega). Purified DNA was sequenced in the forward direction using Sanger dideoxy sequencing technology by a commercial sequencing service (Macrogen, Korea). Sequence analysis was done in BLAST where the query sequence was compared with the sequences deposited in GenBank to find out the closest relative genus/species. The complete 16S rRNA and 18S rRNA ITS sequences have been submitted to the GenBank at the National Center for Biotechnology Information (NCBI).

*(xi) Statistical Analysis.* The statistical analysis was conducted using SAS 9.1.3 software. The data of airborne microbial counts were analysed using GLM (Generalized linear models) procedure. Following two-way analysis of variance (ANOVA), multiple pairwise comparisons were done using post hoc Fisher's LSD test (Least Significant Difference). Probability level below 0.05 (p < 0.05) was set as statistically significant.

## 3. Results

The temperature in the selected study sites ranged from 23°C to 28°C. The number of head counts increased during the hospital visiting hours. The activities in each sampling site varied during the sampling time.

**Figure 1:**
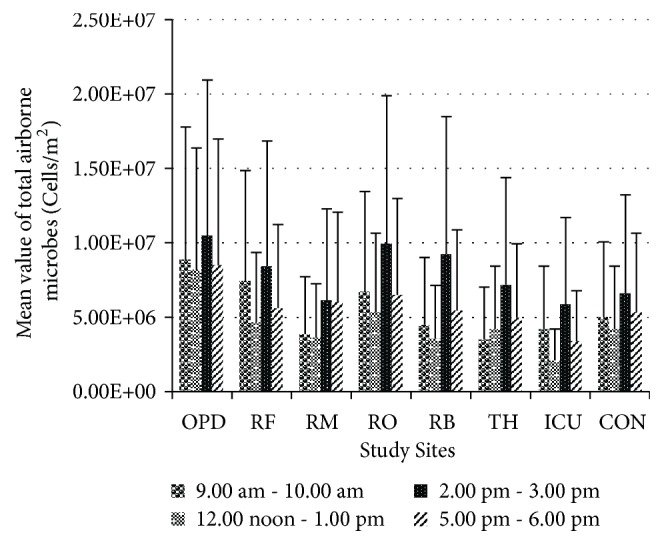
Levels of total airborne microbes in studied sites observed during all four sampling time periods (in cells/m^2^). Data represents the mean of triplicates/three rounds of sampling in each time period with standard deviation. OPD: Outpatients Department, RF: respiratory female ward, RM: respiratory male ward, RO: respiratory diseases unit doctors' staff room, RB: Respiratory Bronchoscopy Unit, TH: Operating Theatre in Gynecology and Obstetrics Department, Surgical ICU: Surgical Intensive Care Unit, and CON: Control.

*(i) Total Airborne Microbial Count.* Statistical analysis between all levels of total airborne microbes showed significant variances (*p*< 0.05) among study sites and sampling time periods. According to the results of mean total airborne microbes in all the sampling periods, the OPD is found to be generally contaminated with mean level of 9.01 × 10^6^ cells/m^2^ followed by respiratory unit–medical officers' room with mean level of 7.12 × 10^6^ cells/m^2^. The surgical ICU showed the lowest level of total airborne microbes which is 3.89 × 10^6^ cells/m^2^. The levels of total airborne microbes in respiratory unit ranged from 4.91 × 10^6^ cells/m^2^ to 7.12 × 10^6^ cells/m^2^. The Operating theatre showed 4.97 × 10^6^ cells/m^2^ which is almost near to the level of respiratory unit–male ward which is 4.91 × 10^6^ cells/m^2^ (Table 1). In addition, a repeated ascending and descending pattern in the levels of total airborne microbes was observed from 9.00 am to 6. 00 pm from all the sites except in surgical ICU (Figure 1). The results indicate significantly higher levels of total airborne microbes in all the study sites during 2.00 pm–3. 00 pm while other sampling time periods did not show statistically significant variances between them. 

*(ii) Culturable Airborne Microbial Count.* A higher number of fungal colonies were observed in almost all the culture plates except from Surgical ICU and Operating theatre during the month of March which included two sampling periods, 2.00 pm–3.00 pm and 5.00 pm–6. 00 pm. This observation was different from other sampling sessions carried out in January, February, and April in which higher number of bacterial colonies was observed compared to fungal colonies.

**Figure 2:**
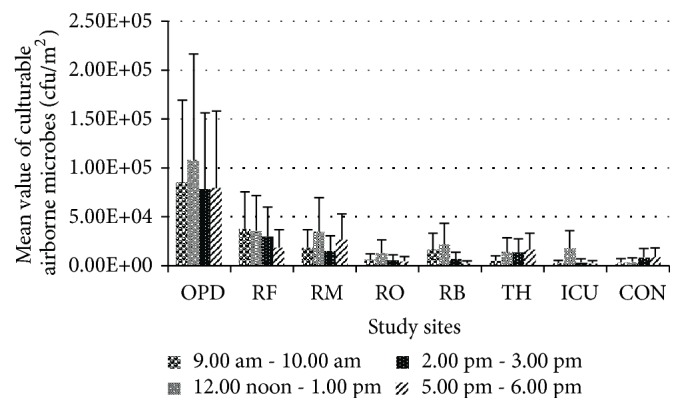
Levels of culturable airborne microbes in studied sites observed during all four sampling time periods (in cfu/m^2^). Data represents the mean of triplicates/three rounds of sampling in each time period with standard deviation. OPD: Outpatients Department, RF: respiratory female ward, RM: respiratory male ward, RO: respiratory diseases unit doctors' staff room, RB: Respiratory Bronchoscopy Unit, TH: Operating Theatre in Gynecology and Obstetrics Department, Surgical ICU: Surgical Intensive Care Unit, and CON: Control.

Statistical analysis between all the levels of culturable airborne microbes showed few significant variances (*p* < 0.05) among study sites, sampling time periods, and the triplicates. The results indicate significantly higher levels of culturable airborne microbes in all the study sites in hospital during 12.00 noon–1.00 pm. According to the results of mean culturable airborne microbes in all the sampling periods, the OPD is found to be generally contaminated with mean level of 8.76× 10^4^ cfu/m^2^ followed by respiratory unit–female ward with mean level of 3.04× 10^4^ cfu/m^2^. The lowest level of airborne microbes was recorded in Surgical ICU within the hospital which is 6.62 × 10^3^cfu/m^2^. The control site showed the lowest level of total airborne microbes which is 6.36 × 10^3^cfu/m^2^ when compared to the hospital in whole. The levels of total airborne microbes in respiratory unit ranged from 7.35 × 10^3^cfu/m^2^ to 3.04× 10^4^cfu/m^2^. The Operating theatre showed 1.24× 10^4^cfu/m^2^ (Table 2). Unlike in the total airborne microbe levels (Figure 1), the graphical representation of culturable airborne microbes level (Figure 2) shows a descending–ascending pattern over the sampling periods depicting the increasing trend in the level of airborne microbes during the visiting hours with few exceptions.

*(iii) Species Identification.* The strains identified by DNA sequencing were the following:

 
*Bacillus sp* (MF480450)
 
*Bacillus cereus* (MF480467)
 
*Bacillus infantis* (MF480460)
 
*Bacillus licheniformis* (MF480451)
 
*Bacillus oryzacorticis* (MF480454)
 
*Brevundimonas vesicularis* (MF480457)
 
*Citrobacter freundii* (MF480464)
 
*Enterobacter cloacae* (MF480461)
 
*Escherichia coli* (MF480448)
 
*Massilia haematophila* (MF480456)
 
*Micrococcus luteus* (MF480452)
 
*Micrococcus sp* (MF480455)
 
*Pseudomonas stutzeri* (MF480446)
 
*Staphylococcus cohnii* (MF480458)
 
*Staphylococcus saprophyticus* (MF480449)
 
*Staphylococcus sciuri* (MF480465)
 
*Staphylococcus succinus* (MF480453)
 
*Aspergillus versicolor* (MF576084)
 
*Fusarium sp* (MF576086)
 
*Trichosporon inkin* (MF576085)

Nine isolates were identified as* Serratia marcescens*,* Bacillus thuringiensis, Exiguobacterium* sp,* Kocuria* sp,* Pseudomonas taiwanensis, Sphingomonas* sp,* Paenibacillus* sp,* Fusarium equiseti*, and* Aspergillus niger* by comparing the morphological characters and Gram staining with microorganisms (*Serratia marcescens* (KT985379),* Bacillus thuringiensis* (KU510061),* Exiguobacterium* sp (KT985374),* Kocuria *sp (KT985360),* Pseudomonas taiwanensis* (KU510064),* Sphingomonas *sp (KT985361),* Paenibacillus *sp (KX641080),* Fusarium equiseti* (KU565728), and* Aspergillus niger* (KU565727)) which were identified by DNA sequencing in a previous study by us.

## 4. Discussion

The present study was carried out at a preliminary level to assess the concentration of airborne microorganisms in a national healthcare setting in Sri Lanka. The total and culturable numbers of airborne microbes in the sampling sites were estimated by fluorescent microscopy and enumeration of colony forming units, respectively. Statistical analyses have shown significant variances among sampling sites and periods in terms of microbial cell counts. The comparison between the two methods of quantification of airborne microbes' level in all the sites including the control site revealed that the total count (Figure 1) estimated was always higher than the culturable count (Figure 2). Another study has reported similar finding in which the airborne microbes in a hospital ward were captured by active sampling and the colony counts recorded 459-1392 cfu/m^3^ while real-time PCR (total count) recorded higher mean levels of* E. coli* 7.37 × 10^7^–1.94 × 10^10^ CFU/m^3^ in the same ward [8]. The results of present study indicate significantly high levels (*p* < 0.05) of total airborne microbes in all the study sites during 2.00 pm–3. 00 pm (Figure 1) and during this period the mean of total airborne microbial levels ranged from 5.85 × 10^6^ cells/m^2^ to 1.05 × 10^7^ cells/m^2^ in the hospital, with theatre showing 7.19 × 10^6^ cells/m^2^ which is almost the same to the counts obtained in respiratory diseases unit. Sampling procedure during this period was carried out from the end of February to mid-March. Similar finding has been reported in an Indian study in which the highest bacterial population (0.25 × 10^2^ cfu/m^3^ to 7.25 × 10^2^ cfu/m^3^) was recorded in the afternoon between 1.00 pm and 2.00 pm compared to morning and evening [9]. However, one study has reported that viable bacterial and fungal counts were higher during the evening period 5.00 pm–6.00 pm compared to morning and afternoon in a Nigerian hospital [10]. Interestingly, one-week period of March showed higher levels of airborne microbes in both total and culturable counts.

The temperature recorded in March was between 23.5°C and 29°C in the hospital and most of the sites in the hospital showed temperature above 26°C in this period while the control site showed 23°C–26°C. Throughout the sampling period, the mid-February–mid-April showed higher temperature readings. In addition, March, 2017, had many rainy days compared to other months. The whole sampling period spent in the hospital coincided with the construction of buildings near the location of operating theatre. Although the dust contamination generated by construction works could be the reason for elevated airborne microbial levels in the theatre it could have been the same condition throughout the sampling period. Studies conducted in countries such as Iran, Korea, and Taiwan revealed higher levels of airborne bacterial and fungal levels during hot summer months with an average daily temperature of 28°C in Taiwan [8, 11, 12]. High humidity levels and condensation during excessive rainfall lead to the absorption of moisture by the building materials which in turn can support microbial growth and increase the settling rate of bioaerosols [13, 14]. According to one study humidity in the hospital environment significantly correlated to airborne bacterial concentration, but not temperature [15]. One study reported that the airborne fungal concentration in a hospital environment was significantly affected by humidity [14]. The fungal spore counts increase during the summer with higher daily temperature both indoors and outdoors [16]. Large number of fungal spores can increase the total counts. Furthermore, the number of fungal colonies observed on the culture medium was higher than the number of bacterial colonies during the sampling period in March. Therefore, it further justifies the incident of sharp rise in airborne microbial levels during this period. However, the findings of studies related to effects of environmental factors on airborne microbial counts are inconsistent. Few studies disproved the effects of environmental factors on airborne microbial levels in the hospital environments [17, 18].

The culturable counts of present study showed significantly (*p* < 0.05) higher mean levels of airborne microbes in hospital environment during 12.00 noon–1.00 pm ranging from 1.32 × 10^4^ cfu/m^2^ to 1.08 × 10^5^ cfu/m^2^. The above-mentioned time period represents the usual hospital visiting hours after 6.00 am–7.00 am in a day. It is apparent that the occupant density of hospital environment increases during hospital visiting hours. In addition to the patients and healthcare workers, the visitors, their activities, and the items brought by them become additional sources of bioaerosols and increased human activity within a short period of time accounts for a sudden rise in airborne microbial level in hospitals [11, 19–21]. The comparison of both total and culturable airborne microbial counts between female and male wards in the respiratory unit revealed higher levels of microbial counts in female ward despite the fact that both of the wards consisted of patients with various respiratory diseases including tuberculosis and lung cancer. This finding is supported by many studies conducted in other countries which show similar results [10, 21–23]. The highest counts of total and culturable airborne microbes in the female ward were 8.42 × 10^6^ cells/m^2^ and 3.77 × 10^4^cfu/m^2^, respectively. The highest counts of total and culturable airborne microbes in the male ward were 6.14 × 10^6^ cells/m^2^ and 3.47 × 10^4^cfu/m^2^, respectively. According to published literature, presence of sex disparities in terms of pulmonary diseases is apparent and a number of pulmonary diseases affect women differently and with a greater degree of severity than men and this is due to biomass burning and other domestic roles of women which expose them to polluted indoor air for longer periods of time [24, 25]. In addition, the fact that the female ward is situated close to the main road can cause large influx of dust particles contaminated with microorganisms through the windows. The absence of exhaust fans in both female and male wards under study can exaggerate the problem as the circulating air from the wards cannot be drawn out. In the absence of exhaust fans, the air within the wards can harbour large number of microbial cells in addition to the microbial cells that enter inside through the windows. According to culturable airborne microbial counts (Table 2), the bronchoscopy unit showed higher counts during operational time (9.00 am–12.00 noon) than at resting/ nonactive time (2.00 pm–6.00 pm). In addition to the facts that the bronchoscopy unit is located facing the road and it is a small space without an exhaust fan, the activities carried out in bronchoscopy unit stood as major sources of bioaerosols such as coughing, ineffective bronchoscopy cleaning procedure, accessories, instilled solutions, and contaminated reprocessing equipment [26–28]. The medical officers' room in the respiratory unit where medical officers rest also showed relatively higher counts of total and culturable airborne microbes. The room was a very small enclosed space without any windows and the air conditioner is the only means of ventilation. The doctors spend more time examining the patients in both female and male wards and carry out bronchoscopy procedures. Therefore, they tend to carry higher number of microbes on their clothes and body parts exposed to air. The aerosolized bacteria from oral, nasal, and pulmonary flora of patient can be inhaled by medical staff [27]. A Brazilian study reported of isolation of potentially pathogenic and toxigenic fungi from healthcare staff who are associated with ICU and operation theatre [29].

The operating theatre and surgical ICU also showed relatively higher counts. Despite the fact that these three sites were equipped with air conditioning systems, higher levels of airborne microbial concentration were detected. It was observed that highest levels in operating theatre and ICU were detected between 12.00 noon and 6.00 pm. Exceeded level of airborne microbes in operating theatre and ICU compared to standard level was reported in a number of other studies conducted in Brazil, Pakistan, Nigeria, Italy, and Iran [12, 17, 29–31]. The operating theatre under study had two main doors and during the sampling it was observed that the doors were frequently opened and the path leading to the main door was wide open most of the times. As earlier mentioned, the sampling time of this present study coincided with a construction work in the hospital. One of the main doors in the theatre was near to the place where the construction work was carried out. Visible dust particles accumulated over a long time period were also observed outside the theatre along with old, broken furniture. Apart from this, the theatre is located close to other units of the hospital. However according to some other studies the operating theatre and ICU showed the lowest microbial counts compared to other wards. The reasons for lower airborne microbial counts in operating theatre and ICU given were high sanitary conditions and good ventilation methods as they are sensitive zones in the hospital [9, 10, 19, 20]. The above findings from both developed and developing countries suggest that the air quality of an operating theatre and ICU depends entirely on the safety protocols and the management of hospital. This statement seems to be supported by another similar study [19]. Few publications on airborne microbial levels include comparative studies between government hospitals and private hospitals. Interestingly, in all of the studies operating theatre, patients ward, and ICU of government hospitals had higher levels of airborne microbial concentration than those of private hospitals [9, 10, 19]. Government hospitals are fully funded by the government and are nonprofit hospitals because the services are free or less charge, which attracts more people especially low income families. Additionally, the government resources have to be allocated equally or according to the needs of all government subsidized hospitals within the country. Therefore, government hospitals face resource constraints in terms of money and technological advancements and it causes low indoor air quality because of heavier human traffic, lack of proper ventilation methods and equipment, and long term usage of infrastructure. A Sri Lankan study has concluded that the Kandy General Hospital is one of the areas with higher level of air pollution in Kandy city [32]. According to another Sri Lankan study the degree of air pollution in Kandy is greater and one of the main reasons of this is the geography of Kandy surrounded by mountain ranges which facilitate transboundary air pollution [33].

The highly pathogenic strains identified by DNA sequencing were* Bacillus cereus *and* Trichosporon inkin*. According to literature,* Bacillus oryzacorticis* and* Pseudomonas taiwanensis *have not been identified as human pathogens. Except these strains all other identified microorganisms are regarded as opportunistic and common nosocomial infectious agents.* E. coli* was isolated in OPD, operating theatre, and respiratory unit–male ward and bronchoscopy unit. Some studies have concluded that* E. coli* is the most abundant species in the hospital air [8, 10]. According to literature it is implicated in urinary tract infections, septicemia, pneumonia, neonatal meningitis, peritonitis, and gastroenteritis [9, 34].* S. succinus*,* S. sciuri*,* S. cohnii*, and* S. saprophyticus* represent one of the major nosocomial pathogens because of their nature of being typical opportunists. They were detected in all the sampling sites. These strains may cause bacteremia relating to catheters, surgical prostheses such as spinal fixation material, acute cholecystitis, endophthalmitis, septic shock, brain abscess, peritonitis, endocarditis, pneumonia, urinary tract infection, septic arthritis, and most frequently wound infections [35–37]. In addition, these strains are resistant to methicillin and semisynthetic penicillin [38].* E. cloacae* has been detected in respiratory unit-male ward and bronchoscopy unit.* E. cloacae* is an opportunistic pathogen and is well known to cause nosocomial infections. According to literature, several incidents of* E. cloacae* outbreak in ICU and theatre have been reported with death occurrences [39–41].* Enterobacter *sp are known to cause meningitis, bacteremia, endocarditis, and infections in lower respiratory tract, skin, bone and joint, urinary tract, intra-abdominal region, and central nervous system.* P. stutzeri *was detected in all sampling sites of hospital. According to literature,* P. stutzeri* has been isolated from several clinical samples from surgical wounds, blood, respiratory tract, and urine and has caused vertebral osteomyelitis, septicemia, and prosthetic infections with death incidents [42–44].* B. vesicularis *was found in the air of all sampling sites within the hospital except operating theatre. According to literature* B. vesicularis* is an opportunistic pathogen and causes nosocomial infections including bacteremia, liver abscess, and meningitis [45–47].* Bacillus *sp,* B. cereus*,* B. licheniformis, B. thuringiensis*, and* B. infantis* were isolated from most of the sampling sites. The ability to form spores enables* Bacillus* species to withstand harsh environmental conditions and show resistance against common disinfection procedure.* B. cereus* is associated with food poisoning leading to fatal gastrointestinal infections, nosocomial bacteremia, pneumonia, wound infections, and central nervous system infections.* Micrococcus *sp and* M. luteus* were isolated from all the sampling sites including control.* M. luteus* is considered to be an emerging nosocomial pathogen.* M. luteus* causes bacteremia, septic shock, septic arthritis, endocarditis, meningitis, intracranial suppuration, and cavitating pneumonia. Few studies reported that* Micrococcus *sp was found to be prominent in hospital environment along with* Staphylococcus *sp [13, 48]. All the sampling sites showed the presence of* M. haematophila* in the air. It has been included in the list of medically important bacteria and has also been isolated from blood culture specimen of which the source of blood is unknown [49].* Sphingomonas *sp and* Exiguobacterium *sp, being nosocomial pathogens, were isolated from the indoor environment of all sampling sites. They are believed to cause bacteremia, skin infections, and septic shock [50]. According to the present study,* C. freundii* has been isolated from OPD and respiratory unit-female and male wards. It is found in environment, animals, and human.* Citrobacter* sp including* C. freundii* cause various nosocomial infections in urinary tract, intra-abdominal region, bone, respiratory tract, endocardium, wounds, soft tissue, meningitis, nose, ear, and bloodstream [51, 52].* Citrobacter *sp is emerging as third most common urinary tract pathogen and it is resistant to commonly available antibiotics [53].* Paenibacillus *sp was isolated from OPD and respiratory unit-female ward and male ward. It has been isolated from neonatal intensive care unit and blood samples of neonates who had nosocomial bacteremia. It was revealed that contaminated rubber stoppers of blood culture bottles were the sources of* Paenibacillus *sp [54]. According to the results of present study* P. taiwanensis *was isolated from OPD. It is a novel bacterium isolated from soil in 2010 [55]. Further details or presence of this bacterium in hospital environments was not documented in the available literature so far.* S. marcescens* was isolated in all the sites except male ward, operating theatre, and control site. It is a well-known nosocomial pathogen that usually causes epidemics in intensive care units. It causes severe high mortality rate infections in respiratory tract, urinary tract, bloodstream, surgical wounds and soft tissues, Meningitis, endocarditis, and osteomyelitis [56, 57]. According to one study* S. marcescens* caused outbreaks of nosocomial bacteremia, sepsis, and meningitis in a special care baby unit in United Arab Emirates which resulted in five deaths. The reservoir of* S. marcscens* was found to be the air flow from the air conditioning systems [58].* K. kristinae* was detected in all the study sites of hospital. It causes urinary tract infections, catheter-associated bacteremia, dacryocystitis, skin infections, peritonitis, brain abscess, and meningitis [59].* K. kristinae* is considered as a true pathogen in pediatric patients [60]. The fungi* A. versicolor, A. niger, F. equiseti*, and* Fusarium *sp have been isolated from all study sites within the hospital and control site.* Aspergillus *sp and* Fusarium *sp are ubiquitous in environment.* Aspergillus *sp causes aspergillosis infection. Aspergillosis is acquired by inhalation of airborne dust particles that carry the* Aspergillus *sp spores; it is followed by pneumonia development and the fungus disseminates through the bloodstream to other organs [9].* Fusarium *sp causes fusariosis infection in skin and eyes and can become an invasive disease in blood [61]. Two studies have reported that higher counts of* Aspergillus* and* Fusarium *sp were detected during the period of construction work in the hospital [29, 62]. The construction work is considered to be the major reason of 49.1% of* Aspergillus* sp outbreak within hospitals [63].* T. inkin* was isolated from operating theatre.* Trichosporon *sp are yeast like fungi and can cause invasive disease [64].* Trichosporon *sp has emerged as an important nosocomial pathogen causing fungemia with a high mortality rate in immunocompromised patients [65].* T. inkin* causes skin infections, onychomycosis, pneumonia, endocarditis, peritonitis, lung abscess, and other invasive infections [65, 66].

Few studies have concluded that hospital environment including clean rooms, ICU, and operating theatre had significantly more bacteria than fungi in the air and surfaces and most of them were related to human body rather than environment [19, 22, 67, 68]. A study says that most of the bacteria found in the air of hospital environment originate from human skin or the gut [28]. According to the results of present study, it is apparent that* Bacillus *sp,* Micrococcus *sp,* Pseudomonas *sp,* Staphylococcus *sp,* Exiguobacterium *sp,* Sphingomonas* sp,* Massilia *sp,* Kocuria *sp,* Fusarium *sp, and* Aspergillus *sp have colonized all the sampling sites within the hospital. It should be noted that all the bacteria (except* B. oryzacorticis* and* P. taiwanensis*) and fungi isolated in present study are identified as highly pathogenic/opportunistic pathogens/medically important microorganisms. Future studies with individual patient characteristics and healthcare staff would help to factor the possible reasons of the existence of pathogenic microorganisms within hospital environment. Viruses are another major cause of nosocomial infections and the future studies aiming on quantification and identification of viral particles would greatly help to determine the air quality within the hospital.

## 5. Conclusion

Studies on aerobiology within the hospital environments would be of great value in helping to assess the air quality and to reduce the incidence of hospital acquired infections. The exposure of patients, healthcare personnel, and visitors to these microorganisms is inevitable. The immune status of persons and the dose of virulent pathogen determine the disease development. The colonized hospital environment with microorganisms is of substantial importance although the main sources of airborne microorganisms are patients, healthcare staff, and visitors. The construction works within the hospital premises pose a major contribution to increased airborne concentration especially fungi.

The hospital environment with relatively high counts of airborne microorganisms cannot be misunderstood as highly risky to enter or get the services as it cannot be concluded that all the microorganisms existing within the hospital environment are pathogenic and virulent. However, in the present study higher counts of airborne microorganisms were reported in the hospital environment including very sensitive zones. The natural air flow cannot be controlled everywhere and the possibilities of dust entering the hospital site are unavoidable. Thus implementing stringent and frequent disinfection procedures, training of healthcare workers on best hygiene practices, well managed surveillance methodology, and installing high efficiency filtration systems could greatly help the authorities to minimize the airborne transmission of infectious pathogens within the hospital. Infectious disease physicians in collaboration with other departments of hospital must initiate appropriate mitigation measures and formulate evidence based policies.

## Figures and Tables

**Table 1:** Levels of total airborne microbial load in each sampling site (cells × 10^6^ /m^2^).

Sampling Sites	9.00 am–10.00 am	12.00 noon–1.00 pm	2.00 pm–3.00 pm	5.00 pm–6.00 pm	Mean value in each site ± SD (whole sampling period)
Outpatients Department	8.89	8.19	10.5	8.49	9.01 ± 1.01
Respiratory Diseases Unit–Female Ward	7.43	4.68	8.42	5.61	6.54 ± 1.70
Respiratory Diseases Unit–Male Ward	3.86	3.63	6.14	6.02	4.91 ± 1.35
Respiratory Diseases Unit–Staff Room	6.73	5.32	9.94	6.49	7.12 ± 1.98
Respiratory Diseases Unit–Bronchoscopy Unit	4.50	3.57	9.24	5.44	5.69 ± 2.49
Operating Theatre in Obstetrics/Gynecology Department	3.51	4.21	7.19	4.97	4.97 ± 1.60
Surgical Intensive Care Unit	4.21	2.11	5.85	3.39	3.89 ± 1.57
Control	5.03	4.21	6.61	5.32	5.29 ± 0.99

**Table 2:** Levels of culturable airborne microbial load in each sampling site (cfu × 10^4^/m^2^).

Sampling Sites	9.00 am–10.00 am	12.00 noon–1.00 pm	2.00 pm–3.00 pm	5.00 pm–6.00 pm	Mean value in each site ± SD (whole sampling period)
Outpatients Department	8.46	10.8	7.82	7.91	8.76 ± 1.41
Respiratory Diseases Unit–Female Ward	3.77	3.58	2.99	1.83	3.04 ± 0.87
Respiratory Diseases Unit–Male Ward	1.84	3.47	1.53	2.64	2.37 ± 0.87
Respiratory Diseases Unit–Staff Room	0.607	1.32	0.553	0.456	0.73 ± 0.39
Respiratory Diseases Unit–Bronchoscopy Unit	1.66	2.16	0.684	0.243	1.19 ± 0.88
Operating Theatre in Obstetrics/Gynecology Department	0.505	1.42	1.37	1.66	1.24 ± 0.50
Surgical Intensive Care Unit	0.263	1.78	0.351	0.251	0.66 ± 0.74
Control	0.363	0.398	0.874	0.909	0.63 ± 0.29

## Data Availability

The data used to support the findings of this study are available from the corresponding author upon request.
